# Health Challenges of the Pacific Region: Insights From History, Geography, Social Determinants, Genetics, and the Microbiome

**DOI:** 10.3389/fimmu.2019.02184

**Published:** 2019-09-13

**Authors:** Paul F. Horwood, Arnaud Tarantola, Cyrille Goarant, Mariko Matsui, Elise Klement, Masahiro Umezaki, Severine Navarro, Andrew R. Greenhill

**Affiliations:** ^1^College of Public Health, Medical and Veterinary Sciences, James Cook University, Townsville, QLD, Australia; ^2^Institut Pasteur de Nouvelle-Calédonie, Noumea, New Caledonia; ^3^Internal Medicine and Infectious Diseases Department, Centre Hospitalier Territorial, Noumea, New Caledonia; ^4^Department of Human Ecology, Graduate School of Medicine, The University of Tokyo, Tokyo, Japan; ^5^Immunology Department, QIMR Berghofer Medical Research Institute, Herston, QLD, Australia; ^6^School of Health and Life Sciences, Federation University Australia, Churchill, VIC, Australia

**Keywords:** Pacific, Oceania, microbiome, nutrition, genetics, infectious disease, non-communicable disease

## Abstract

The Pacific region, also referred to as Oceania, is a geographically widespread region populated by people of diverse cultures and ethnicities. Indigenous people in the region (Melanesians, Polynesians, Micronesians, Papuans, and Indigenous Australians) are over-represented on national, regional, and global scales for the burden of infectious and non-communicable diseases. Although social and environmental factors such as poverty, education, and access to health-care are assumed to be major drivers of this disease burden, there is also developing evidence that genetic and microbiotic factors should also be considered. To date, studies investigating genetic and/or microbiotic links with vulnerabilities to infectious and non-communicable diseases have mostly focused on populations in Europe, Asia, and USA, with uncertain associations for other populations such as indigenous communities in Oceania. Recent developments in personalized medicine have shown that identifying ethnicity-linked genetic vulnerabilities can be important for medical management. Although our understanding of the impacts of the gut microbiome on health is still in the early stages, it is likely that equivalent vulnerabilities will also be identified through the interaction between gut microbiome composition and function with pathogens and the host immune system. As rapid economic, dietary, and cultural changes occur throughout Oceania it becomes increasingly important that further research is conducted within indigenous populations to address the double burden of high rates of infectious diseases and rapidly rising non-communicable diseases so that comprehensive development goals can be planned. In this article, we review the current knowledge on the impact of nutrition, genetics, and the gut microbiome on infectious diseases in indigenous people of the Pacific region.

## Introduction

The Pacific region is a loosely defined group of countries and territories that share a border with the Pacific Ocean. The region, sometimes referred to as Oceania, is diverse in its cultures, ethnicities, economic development, and living standards. Reflecting this diversity, health indicators vary considerably across the region, with life expectancy ~20 years higher and infant mortality up to 15 times lower in the best performing countries (Australia—life expectancy: 82.8; infant mortality: 4/1000 live births; and New Zealand—life expectancy: 81.6; infant mortality: 6/1000 live births) relative to the poorest performing countries (Papua New Guinea—life expectancy: 62.9; infant mortality: 57/1000 live births; and Solomon Islands—life expectancy: 69.2; infant mortality: 28/1000 live births) (1).

Although indigenous populations in the Pacific have developed complex stratagems and adapted highly effectively to their sometimes extreme environment, Australian Aboriginal & Torres Straits Islanders, Micronesian, Melanesian, and Polynesian populations are overrepresented among severe cases and deaths related to certain diseases, both communicable and non-communicable (2–5). A partial explanation of this over-representation is the challenges faced in many Pacific island countries in the delivery and uptake of: health services, improved water and sanitation, and education; problems faced in many low-income countries globally. However, factors specific to the region and its people, such as rapidly changing diets and the historic isolation of these ethnic groups, need also be considered.

Studies conducted on the relationships between host genetics, microbiome, and diseases are mainly carried out in Europe, Asia, or USA, and include very few or no indigenous participants from the Pacific despite the high burden of some diseases they face. Conclusions drawn from studies conducted in other geographical areas determine global trends, but have little specific application to Pacific populations (6–8), leading to a dearth of knowledge on how the microbiome may impact upon and/or interact with susceptibility to disease and immunomodulation.

Despite current limitations, numerous studies have investigated the ethnography and relatedness of the Pacific people. Data exists on burden of diseases in Pacific nations, and to a lesser extent in Pacific migrants living overseas. Using these data and drawing on primarily international literature on immunomodulation and the gut microbiome; we explored these key factors and their role on health and disease in Pacific people.

## Historical Peopling of the Pacific

**Figure 1:**
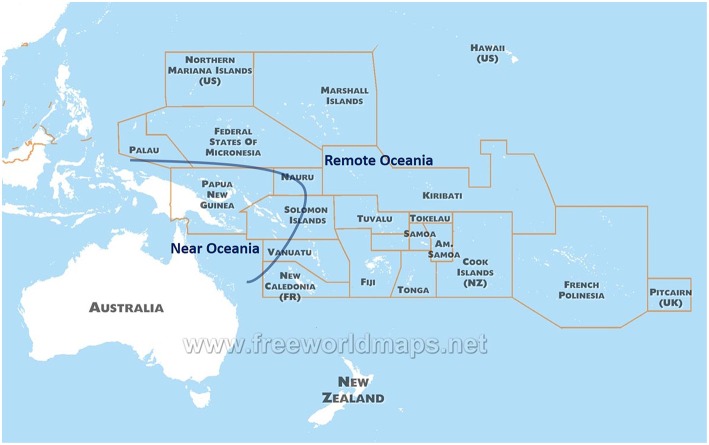
A map of the countries and territories of Oceania.

Indigenous populations in the Pacific arose from the last great human migration into an uninhabited continent, and differ genetically from other populations present across the Globe. The Pacific has two distinct regions in anthropology: Near Oceania (Australia, New Guinea Island, and Solomon Islands) and Remote Oceania (to the east of Solomon Islands, including up to Easter Island and Hawaii Islands) (9) (Figure 1). Archaeological evidence suggests human settlement in Near Oceania around 47,500–55,000 years before present (10). Recent sequence analysis of genomes from indigenous groups in the Pacific has revealed that ~3–6% of DNA from Melanesian and Indigenous Australian people derive from an ancient human lineage called the Denisovans (11, 12). This DNA was possibly introduced during the early migration to Near Oceania, and further illustrates the historic isolation and uniqueness of the Pacific people.

Human settlement of Remote Oceania occurred only within the past 3,100 years: New Caledonia 1,200 BC, Tonga 900 BC, Samoa 800 BC, Hawaiian Islands 900 AD, and New Zealand 1,200 AD (13). The indigenous people of Remote Oceania probably originated from the East Asian region, in particular the Southern regions in China or Taiwan. This is supported by linguistic evidence as indigenous populations in Taiwan and Remote Oceania both speak Austronesian languages (14). Genomic studies also suggest that there was admixture between East Asian and Near Oceania ancestry. However, it is still debated where the admixture occurred: at the northern coastal region of the New Guinea Islands while East Asian ancestry moved toward Remote Oceania (9); or in Remote Oceania, where Near Oceania ancestry later arrived and mixed with East Asian ancestry (15).

Interestingly, in the context of this review and the impact of gut microbiome on health (below), supporting evidence of the two waves of migration in the region comes from phylogenetic study of *Helicobacter pylori;* a pathogen associated with stomach ulcers that was present in human populations during the human migration out of Africa and has been previously used to provide insights into human migrations (16, 17). Haplotyping of *H. pylori* in indigenous populations in the Pacific reflects the time since divergence, with the *H. pylori* hpSahul haplotype associated with indigenous people living in Near Oceania diverging from an Asian haplotype some 20,000 to 25,000 years before the *H. pylori* hspMaori haplotype isolated from indigenous people living in Remote Oceania diverged (18).

Both populations in Near Oceania and Remote Oceania are assumed to be historically isolated from other populations; as highlighted by over 800 language groups in Papua New Guinea (19), and also supported by *H. pylori* phylogenetics (18). The populations in Near Oceania rarely interbred with neighboring populations in the past. For example, the endogamy rate in the 1940-50s in both the Kombio-speaking group (Sepik, Papua New Guinea) and the Gidra-speaking group (Oriomo Plateau, Papua New Guinea) were >95% (20, 21). Indigenous populations in Remote Oceania were geographically isolated on islands that were separated by vast distances. However, genetic, linguistic, and archaeological evidence suggests that the Polynesian people of Remote Oceania maintained a complex trade network that extended from Hawaii in the north, New Zealand in the south, and Easter Island in the east (22, 23)—the so-called “Polynesian triangle.”

## Demographic and Health Indicators in the Pacific

The Oceanian territories (land mass) in the South Pacific total about 8.5 million km^2^ (adapted from Pocket Statistical Summary: https://prism.spc.int/) over a total area of about 80 million km^2^ (estimated using GoogleEarth Pro: https://www.google.com/earth/). It is populated by ~41 million inhabitants, although approximately two-thirds of those people live in Australia and New Zealand, where European settlement accounts for the majority of the population and has had major cultural and socio-economic impacts. There are ~12.3 million inhabitants of the Pacific that have whole or partial indigenous Oceanian origins [details of the demographics of indigenous people in the Pacific are reviewed in (24)]; including the Indigenous Australian (Aboriginal and Torres Strait Islanders) and New Zealand Maori populations. This distinction between indigenous Oceanians and descendants of immigrant ancestries (arrived within the past 230 years) is useful in a public health context, because health indicators differ greatly, reflecting both the differing challenges and vulnerabilities faced by population subgroups, and access to healthcare and education.

The Pacific consists of many countries and territories that are geographically small and isolated, with comparatively small populations. This has consequences in terms of health delivery, but also on the local impact on global health priorities and the ability to attract international awareness and subsequent funding. To this end, there is some value in considering the total population of indigenous Oceanians, which is comparable in number to that of South Sudan, Rwanda, Somalia, or Chad. These countries are located at or near the 67th percentile of populations in countries/territories (25) and have health indicators that are broadly comparable to Papua New Guinea (the most populated Pacific country of primarily indigenous people), but appear to be a higher priority in global health.

Importantly, while the collective population of the Pacific is comparable to a moderately populated country, it is undergoing rapid growth. According to United Nations projections, the total population of Oceania (including Australia) will reach about 47 million in 2030 and 57 million in 2050 (25). Much of this growth is predicted to come from countries such as Papua New Guinea and other countries of predominantly indigenous populations, and will put further pressure on already challenged health systems.

## A high Burden of Disease Among Indigenous Populations in the Pacific

Indigenous Oceanians face many of the health challenges identified in similar low-income settings, though cultural and biological constructs associated with health and disease differ. There is relatively abundant and well-documented data to suggest that certain health issues disproportionately affect Maoris in New Zealand, Polynesians in Hawaii and the Aboriginal & Torres Straits Islanders in Australia compared to their non-indigenous counterparts in the same countries (2–4, 26). The incidence of infectious diseases is strongly conditioned by social determinants of health (food and nutrition, housing, sanitation, prenatal stress, etc.), the less favorable of which are often concentrated among the most marginalized populations (5, 27–33). The respective roles of the social determinants of health (education, food, poverty, etc.), genetics and microbiota in the incidence of these diseases among indigenous populations in the Pacific, however, remain to be determined.

In the age of personalized medicine, genetics becomes an important consideration. The majority of known gene variations that distinguish one human group from another are not associated with diseases but display comparable functionality at the clinical level and only illustrate human genetic diversity (34). Identifying genetic vulnerabilities among populations can be very important for medical management (6, 35). For example, the identification of a P2X7R polymorphism has been associated with an increased risk of tuberculosis in mice (36) and later a Tibetan Chinese population (37). Further studies have suggested new therapeutic avenues through the use of natural agonists to P2X7R that promote death of *Mycobacterium tuberculosis* and apoptosis of infected monocytes and macrophages (38).

In addition to pre-existing immunity, vulnerability to a particular disease is partly due to a population not having been exposed for a long time to the pathogen that causes it. Specific vulnerabilities can arise from genetic characteristics selected for the advantage they confer in the face of another, more prevalent or severe infectious risk (39, 40). Certain pathologies linked to genetic characteristics may also be more frequent in some subgroups, mainly because of the emergence of recessive traits due to human reproduction in isolated settler populations; whether these are isolated from a geographical or environmental standpoint, or for cultural, linguistic or religious reasons. In the global context an example of such a disease is Tay-Sachs disease among Ashkenazi Jews, with the recessive carriage rate (1 in 30) approximately 10 times higher in Ashkenazi Jews compared to the general population in United States (39, 41).

Indigenous populations in the Pacific, however large and geographically scattered, could derive from a very small number of surviving individuals at some point in history (genetic bottlenecks), because groups were small and/or possibly faced with extreme conditions, including those encountered in central Australia, Papua New Guinea or during long transoceanic migrations (18, 42–44). This “perilous travels theory” is disputed by some (45, 46); but is difficult to discount as being a factor in at least some populations in the Pacific.

Global extrapolations supported by limited geographically specific data suggest that genetic selection pressures could, in part, explain the significantly higher burden of some diseases in indigenous populations in the Pacific. The interactions between genetics and burden/susceptibility for infectious and immune mediated diseases such as influenza and rheumatic fever, and non-infectious diseases such as obesity and type 2 diabetes, likely contribute to the health of Pacific people. Selected infectious and immune-mediated diseases are discussed below based on their disproportionate burden in indigenous populations in the Pacific and the emerging lines of research that may help to explain their impact in these populations.

### Influenza

Indigenous populations in the Pacific suffered a disproportionately high mortality during the influenza pandemic of 1918–1919 (47–52). While it is estimated that 3% of the world's population died of influenza during the 1918 pandemic, mortality reached 22% in some Pacific nations such as Western Samoa (50, 51), and 50% in some Australian Aboriginal communities (53). The mortality rate in indigenous Hawaiians was also four-times higher than their non-indigenous counterparts (54). Although the factors that underlie these excess mortalities remain debated (55–59), recent evidence suggests that modern indigenous populations in Pacific Island countries (49, 60–71) are overrepresented 2- to 5-fold among the severe forms of influenza virus infection, such as during the 2009 influenza pandemic in New Caledonia (62, 68). Furthermore, between 2002 and 2014 (excluding 2009) the rate of reported influenza was up to 6.4 times higher among Australian Aboriginals compared to non-Aboriginals; the hospitalization rate for influenza was up to 3.5 times higher, and death rate up to 5.5 times higher (72). This is not only true for pandemic influenza: in 2016, the incidence of seasonal influenza in New Zealand was lower among Maori compared to “Europeans or others” (40 vs. 64 per 100,000). However, the incidence of hospital admissions for severe influenza was 2.5 times higher in the Maori population during that period (73).

The clearly documented vulnerability to severe influenza among indigenous populations in the Pacific may be due to the higher prevalence of identified comorbidities (e.g., diabetes, obesity) (62). It may also be due to lower population immunity or immunization coverage, a more adverse socio-economic context, differential access to care, or other factors. However, genetic and microbiotic factors have also been theorized to play a major role (40, 47, 74, 75). For instance, following the 2009 H1N1 influenza pandemic, researchers identified multiple genetic polymorphisms in diverse populations linked with an increased risk to the H1N1 pandemic virus (76–79).

### Rheumatic Heart Disease

Acute Rheumatic Fever (ARF) is an immune syndrome that can occur following an infection with group A *Streptococcus* (GAS). The related aberrant inflammatory response can lead to permanent heart damage in about 60% of cases, resulting in the development of a chronic Rheumatic Heart Disease (RHD) (80). The occurrence of ARF and consequent RHD remain a major public health problem in vulnerable populations (81, 82). In 2015, it was estimated that RHD affected more than 30 million people worldwide each year with more than 300,000 related deaths (81).

RHD is mainly diagnosed in children and adolescents. It is seldom found in developed countries but remains common in emerging countries, particularly in Asia, Africa, and Oceania (80, 83–86), where nearly 84% of the global RHD cases are documented (87–91).

Indigenous populations in the Pacific present some of the highest rates of RHD in the world. In 1985, one study estimated RHD prevalence at 800 per 100,000 in Polynesia, while in New Zealand this risk was 650 per 100,000 among Maori (compared to 90 among non-Maori) (92). In Australian Aborigines in remote rural parts of Northern Territory the incidence was 651 per 100,000: at the time described as the highest incidence of RHD in the world (93). Rheumatic heart disease is also frequently described among Melanesian and Polynesian populations in New Caledonia (94–98).

Epidemiological studies have shown that socio-economic and environmental conditions, including access to healthcare systems, remain among the major determinants of ARF/RHD prevention (98–100). Moreover, several GAS strains identified as “rheumatogenic” were associated with ARF and differentially influence host immune response (101, 102). Genetic susceptibilities of the host should also be taken into account when the immune response to an infectious agent is being characterized. Recently, a meta-analysis of several published case-control studies showed a correlation between polymorphisms on several genes encoding for cytokines and predisposition for RHD (103–106). Studies in Europe have associated RHD to particular HLA (107–109), however, HLA results were inconsistent, and two recent genome-wide association studies (GWAS) conducted in Oceanian populations and European ancestry individuals showed no significance with the risk of developing RHD or ARF, respectively (110, 111). Interestingly, a novel susceptibility signal was identified in the immunoglobulin heavy chain (IGH) locus (110).

### Dengue

Dengue is the most emergent vector-borne viral disease worldwide (112). The behavior of mosquitoes and the circulation of dengue viruses may be profoundly affected by global climate change, to which territories in the Pacific are particularly vulnerable.

Between 2001 and 2008, dengue epidemics in four Asian countries (Cambodia, Malaysia, Philippines, and Vietnam) caused >1 million reported dengue cases, of which nearly 5,000 (0.5%) died. During this same period, dengue epidemics described in six Pacific territories (Polynesia, New Caledonia, Cook Islands, American Samoa, Palau, and Federated States of Micronesia) caused ~50,000 cases, of which 34 (0.07%) died (113). Other available data also suggest a comparatively lower reported case-fatality rate for dengue in the Pacific (114–116), despite genetically similar viruses circulating in Asia and the Pacific (114).

The relatively lower frequency of deaths due to severe dengue in the Pacific with a high proportion of indigenous populations compared to Asian countries could be due to many biases, including: (i) undefined differences in the virulence of strains circulating in Asia and in the Pacific; (ii) underreporting of dengue cases in Asia and the overrepresentation of severe cases admitted to hospital; (iii) better health in general and better hospital care in particular in the Pacific countries in which these studies were conducted. Conversely, studies have correlated dengue severity with obesity and diabetes (117), both of which are highly prevalent in the Pacific and in indigenous populations especially (118–120). Whether the associations are causative or the result of confusion bias in lower socio-economic groups vulnerable to both dengue and obesity/diabetes remains to be determined.

Recent genome wide association and admixture mapping studies have investigated the natural protection and susceptibility for severe forms of dengue infection. Research teams have identified at least seven single nucleotide polymorphisms that are associated with the risk of severe outcomes during dengue infection (121–123). Although, more detailed understanding of the genetic mechanisms related to susceptibility of dengue infection is needed, associations based on ethnicity have been identified that reveal that people with African ancestry are best protected against severe dengue, while Asian and European populations are more susceptible (124, 125). To date, this has not been investigated in the Pacific to our knowledge.

### Leptospirosis

Leptospirosis is a zoonotic disease that most frequently occurs following an environmental infection with pathogenic *Spirochaetes* from the genus *Leptospira*. The infection can range from asymptomatic in most cases (126) to severe life-threatening disease with a high case-fatality rate (127).

Despite the large spectrum of clinical forms, very little research has focused on the host genetic susceptibility to *Leptospira* infection. Following an outbreak in triathletes in Illinois, the first study on genetic susceptibility associated with leptospirosis evidenced the HLA-class II HLA-DQ6 as a significant risk factor (128). However, further studies in independent populations did not confirm this finding but rather identified HLA-class I and the ancestral HLA haplotype (A1, B8, DR3) as well as several SNPs in innate immune genes as conferring susceptibility to leptospirosis (129, 130). Globally, there is still conjecture, reflecting a lack of studies with a significant number of well-defined confirmed leptospirosis cases. Notably, none of the few studies reported to date have included people of Oceanian ancestry. Whether factors from skin or mucosal microbiota contribute to the initial phase of *Leptospira* infection remains to be determined but would deserve further studies (131).

Leptospirosis mostly impacts tropical rural or suburban young active males from vulnerable populations worldwide, imposing a global burden similar to that of schistosomiasis, leishmaniasis, or lymphatic filariasis (132). Of note, Pacific islanders pay the highest toll to this neglected disease by far, with a morbidity rate 10 times higher than global figures (132, 133). Although social and environmental determinants might contribute to this high burden, more research attention should be paid to population-specific genetic and microbiotic factors contributing to this high burden.

## Indigenous Diets in the Pacific and the Transition to a Western Diet

Indigenous diets in Near Oceania are contrasted between the Papua New Guinea highlands and other regions. The Papua New Guinea highlands is believed to be one of the global origins of agriculture, with taro (*Colocasia esculenta*) and banana (*Musa* spp.) domesticated in the area 10,000 and 7,000 years before present, respectively (134). Banana and taro were the staple foods in the Papua New Guinea highlands before the introduction of sweet potato from Central America ~300 years ago. The intensified agricultural system has enabled continuous cultivation of sweet potato production, which has supported high population density in the region. Pigs pigs which are used as bride price or for compensation of deaths during tribal fighting (135), are reared by feeding sweet potatoes, but the nutritional contribution of pig meat is scarce. Studies in Papua New Guinea have reported that sweet potatoes accounted for over 70% of the total energy intake and that only a limited amount of animal protein was consumed (136).

In the non-Highlands regions of Papua New Guinea and Solomon Islands, shifting cultivation of tubers (taro, yam), banana, sugarcane, and leafy vegetables has been conducted. This type of agricultural system has been formulated on the basis of the crops and agricultural technologies introduced by the Austronesia people a few thousand years before, probably mixed with crops domesticated in the Papua New Guinea Highlands. In some parts of these regions, starch extracted from the stem of the sago palm, which is naturally fermented using traditional practices, is consumed as a staple food (137, 138). Since the sago starch contains only small amounts of nutrients other than carbohydrate, hunted animals, and gathered wild plants are also consumed. In coastal areas, aquatic animals such as fish and crustaceans are also commonly consumed.

The islands in remote Oceania can be mostly categorized into two groups depending on how they were formed (139). One group of islands were formed by volcanic activity (“volcano islands”), while the other group of islands were formed when a coral reef rose (“reef islands”). The volcano islands have relatively larger space, higher mountains, and richer fauna/flora than reef islands. This impacts on the abundance and availability of many crop foods.

Protein deficiency has been confirmed by a number of studies in the Pacific region (140). A recent study found that over 80% of the population in a remote region of the Papua New Guinea highlands had protein intakes below the estimated required level (141). Individuals whose protein intakes are below the required level are expected to show clinical signs of protein deficiency, such as decreased muscle mass (142). Yet despite their apparently deficient protein intake people in the Papua New Guinea highlands rarely exhibit such clinical signs (143). Some researchers have speculated that the Papua New Guinea highlands people have biologically adapted to a low-protein diet (144). Indeed, a recent study found evidence of nitrogen fixation in the gut microbiota of Papua New Guinean people (145). However, no evidence was found that nitrogen fixation substantially contributed to the host nitrogen balance.

During the period before colonization, the people who resided in Oceania were almost completely free from obesity (146). The people consumed a low-energy-density diet and engaged in relatively high physical activity for their subsistence, which resulted in a balance between energy intake and energy expenditure (147). Signs of a “nutritional transition” were first observed in Remote Oceania, then in the coastal and islands region of Near Oceania and finally in the Highlands region of Near Oceania (148). During the period of nutritional transition, the people came to consume energy-dense foods imported from Australia and New Zealand (e.g., rice, canned fish, canned meat, lamb/mutton flaps, vegetable oil, beef tallow, and instant noodles). The nutritional transition occurred more rapidly in the urban areas in each region.

**Figure 2:**
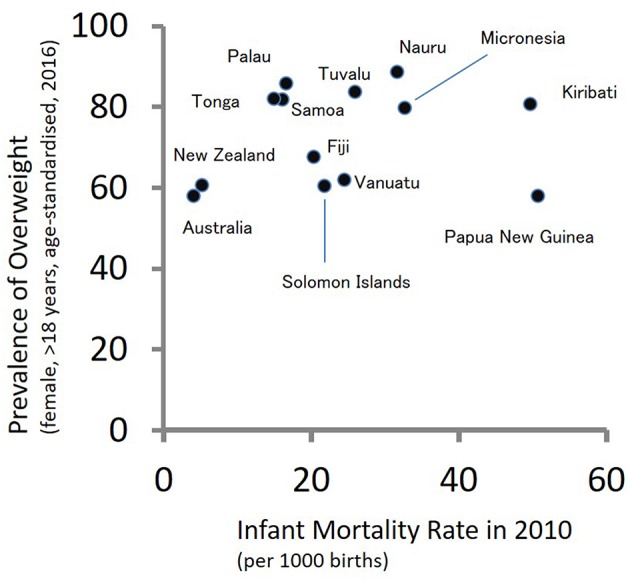
The double burden of infectious and non-communicable diseases in Oceania: a scatter plot between infant mortality rates in 2010 and age-adjusted prevalence of overweight adult females (>18 years of age) in 2016.

According to the theory of epidemiological transition (149), people in subsistence societies before the initial stage of industrialization are faced with high burdens of communicable diseases. As food security, public health, and medicine improve due to industrialization, the burdens of communicable diseases are replaced with those of non-communicable diseases. During the period of epidemiological transition, mortality rates decrease drastically followed by fertility decline. The South Pacific countries have not shown typical patterns of epidemiological transition. Figure 2 shows a scatter plot between infant mortality rates in 2010 and age-adjusted prevalence of overweight adult females (>18 years of age) in 2016 for 13 countries in the South Pacific. A high infant mortality rate reflects the burdens of communicable diseases, while the prevalence of overweight individuals reflects the burdens of non-communicable diseases. As shown in Figure 2, several South Pacific countries such as Papua New Guinea, Kiribati, Micronesia, Nauru, and Tuvalu have high burdens of communicable and non-communicable diseases simultaneously. Such double burdens will provide considerable challenges to the health sectors of these countries.

Oceanians are the most exposed, after the populations of sub-Saharan Africa, to the loss of disability-adjusted life years (DALYs) because of metabolic diseases. Indeed, Oceania residents (all genders and all populations) had the largest increase in BMI in the world for the period 1980–2008, the average BMI in Nauru being the highest in the world in 2008 (118). The link between obesity/inflammatory status (150, 151) and obesity/excess mortality (152, 153) is well-documented, both among Pacific populations and on other continents. After adjustment for age, the prevalence of type 2 diabetes mellitus (T2DM) in the South Pacific was the highest in the world (15–16%). This prevalence increased most strongly in the world between 1980 and 2008 (154), and it is expected that this prevalence will continue to increase in some areas of the Pacific (155). The prevalence of age-adjusted or unadjusted T2DM is highly variable among indigenous Oceanian populations, and can reach 20–30% of the population in some studies (156). By way of comparison, it was estimated in 2012 at about 5.5% of the population in France, and at least 9.4% in the US in 2017 (157, 158). In a New Zealand study, the prevalence of T2DM in Polynesian participants was 2–2.5 times that of participants of European origin (159). The incidence of T2DM was 4–8 times higher among Australian Aborigines than in the general population or compared to the non-indigenous population (160, 161). A review of T2DM in global aboriginal populations found varying prevalence of T2DM between different indigenous groups, which was hypothesized as at least partially due to genetic susceptibility (162). Indeed, a genetic polymorphism in the *HNF1A* gene (G319S mutation) has been identified in the native Canadian Oji-Cree population that was strongly associated with an increased risk of T2DM (163).

## The human gut microbiome

### Pacific Microbiome Relative to a Global Microbiome

Great advances in our understanding of the human microbiome have been made over the past two decades, though the field is arguably still in its infancy. Much of the focus of the human microbiome has been on intestinal microflora and its associated genes; with the current and broadly accepted theories stating that the gut microbiome has the potential to impact greatly on human health and disease. Indeed, most of the microbiome data reported to date are solely focused on bacterial species and their interactions with the human host. Further insights will be gained through elucidating the impact of commensal viruses, archaea, fungi, and protozoans on human health.

The composition of the gut microbiota evolves from birth, with the greatest change occurring at around the age of weaning. By the age of 2 years the gut microbiota of a child resembles that of an adult (164–166), though with some ongoing compositional differences to adult microbiota throughout adolescence (167). Once established, the adult gut microbiota is considered stable, yet subject to perturbation.

Limited studies exist on the gut microbiota/microbiome in the Pacific. The first culture independent study to characterize the gut microbiota in the region used targeted reverse transcription quantitative PCR (168). This approach is likely to be adequately accurate in determining relative numbers (169), but is considerably limited in scope of microbes detected. Nonetheless, findings were indicative of broad similarities between the microbial composition of the gut of PNG study participants and those in other low-income countries, particularly those with some traditional aspects of diet intact as found in a study into the microbiome of people from a rural African village in Burkina Faso (170, 171).

A subsequent study by Martinez et al. (172) used 16S RNA sequencing to investigate the gut microbiota of people in PNG (rural, traditional setting), and compared the composition to study participants in USA (urban Nebraska). This study supported the observation made previously by Greenhill et al. (168) that the microbial composition of people in Papua New Guinea had similarities to other subsistence dwellers (166, 170, 172). Specifically, the presence of a high ratio of *Prevotella* relative to *Bacteriodes*, which is common in people consuming traditional diets where there is a high dependence on fibrous, plant-derived foods (170) found in the Pacific (above).

Microbiome studies conducted in the Pacific, albeit only a small number to date, have helped inform theories of global diversity and distribution of bacteria and mobile genetic elements of the human digestive tract. Global microbiota studies consistently support the notion of greater diversity, or at the very least additional lineages, in populations living a traditional (non-western) lifestyle relative to people in industrialized countries (172, 173). Based on comparison of gut microbial composition of people in Papua New Guinea to people living in USA, Martinez and co-workers proposed that diversity could be explained by the impact of lifestyle on ecological assembly processes. In particular, aspects of traditional life such as diet and lack of sanitation and hygiene likely impact microbial composition.

In addition to the extant lineages associated with people living a traditional lifestyle (including those in the Pacific) and the resulting diversity of the microbiota, one study has investigated the distribution of mobile genes in a Pacific population and compared it to a Western population. In some aspects, the findings were similar to those for bacteria. Most mobile genes were present in some (62%) samples, akin to a core microbiota. Mobile genes for starch hydrolysis were higher in samples from agrarian Fijians than from USA participants, akin to a high *Prevotella* to *Bacteriodes* ratio. The presence of mobile genetic elements was said to be largely independent of species community composition (174).

To date, it has not been possible to elucidate the full composite mechanism that determines gut microbial composition. Ethnicity has some association with the gut microbiota, with Brooks et al. (175) suggesting that 12 taxa of bacteria reproducibly vary across four ethnicities. While conceivably a factor, heritability (thus ethnicity) appears to be a relatively minor influence on microbial composition (176). Indeed, immigration from a non-Westernized country to a Westernized country results in loss of diversity and associated function in the gut microbiome (177). Diet is undeniably an important factor, with people consuming a traditional diet consisting of starch-and fiber-rich foods having bacterial species and mobile genetic elements that reflect those dietary trends. Non-Westernized populations, including Pacific people, are likely to be a niche for extant species that are no longer detected in Western people (178).

### Gut Microbiome, Genetics and Non-communicable Diseases

The prevalence of non-communicable diseases affecting affluent countries has prompted research efforts to focus on better understanding the immunomodulatory role of the microbiome (179, 180). Various studies have sought correlations between lost lineages and “Western diseases” such as obesity, autoimmune, and allergic diseases. However, this is likely an over-simplification of both the etiology of non-communicable diseases and the global burden of disease. Non-communicable diseases are an increasing burden in low-income countries, with heart disease, diabetes, and asthma among the leading 10 causes of all-age disability adjusted life years in the Pacific region (181).

One of the greatest health problems facing Pacific people is obesity, and interventions are urgently needed. The genetic selection of “thrifty genes” (182) has been theorized for indigenous people of the Pacific as a mechanism to cope with food shortages. Now, in the affluent sections of Pacific communities where food shortages are rare, this adaptation could contribute to obesity and type 2 diabetes. The thrifty gene mechanism has been explored in Pima Indians (from the Gila River Indian Community in south central Arizona), through a community-wide longitudinal study running since 1965, and found that the prevalence of T2DM decreases in those who report mixed ethnic ancestry (183–185). The thrifty gene theory remains disputed and seems to insufficiently explain the few data available on indigenous populations in the Pacific (45).

It may be that host genes play less of a role than the collective genes of the microbiome. The notion of an obese microbiome is well-established through both observational studies and transplant studies in animal models (186–189). Similarly, a role of microbiota in diabetes has been investigated, and characterization of the digestive metagenome predicts the presence of type 2 diabetes more robustly than body-mass index (BMI) in women in Sweden and China (190). Further research could help develop new and tailored approaches for post-prandial glycemic control (35, 191).

### Immunomodulation of the Microbiome and Influence on Immune Health

**Figure 3:**
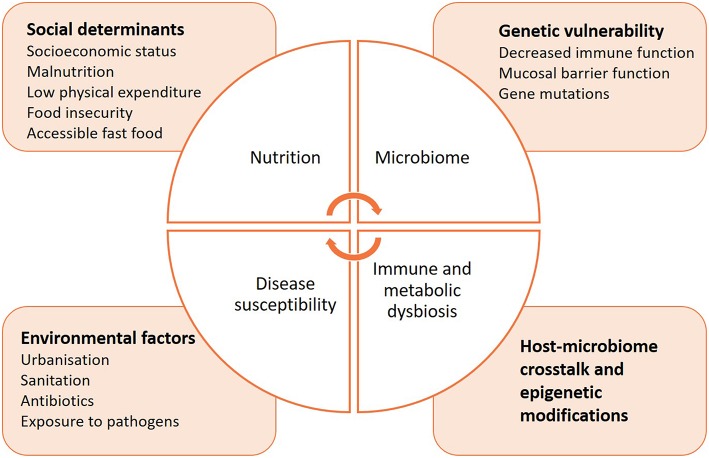
Intersections between nutrition, microbiome, immunity, and susceptibility to infectious diseases.

In comparison to non-communicable diseases, much less is known on how the microbiome may influence the course of communicable diseases, or increase susceptibility to infection (Figure 3). The delicate microbial ecosystem colonizing the human host fosters cooperative interactions with the immune system that is likely to influence how it responds to pathogenic assaults (192). Indeed, microbes synthesize short-chain fatty acids (SCFAs) and other metabolites, 95% of which are readily absorbed by the host (193–195). They can directly alter immunity, metabolism and reproductive functions, and even induce epigenetic modifications (196–201). Animal studies have facilitated the characterization of SCFAs, but the identification of specific bacterial species composing the human host responsible for similar activities has proven difficult (202). A possible reason is that the microbiome composition differs by geographic location due to differences in diet, lifestyle, and environmental exposures. Interestingly, a recent study highlighted the importance of considering the complex dynamics of the microbiota to understand how it affects immunity (166, 171, 203). Commensal viruses and fungi have a critical role in modulating bacterial metabolic pathways and therefore influence their release of specific SCFAs, which may provide a key element in profiling the microbiome in health and disease (204–207). In addition to categorizing the taxonomic diversity of the bacterial communities by sequencing to correlate a specific immune phenotype, the metabolomic signature of the microbiome should be determined, as it best determines the functional role of the microbial ecosystem and its influence on immunity.

It is thought that the gut microbiota can have a negative impact on immune function, thus leading to increases in immune disorders. This led to the development of the hygiene hypothesis (208), which has evolved into more nuanced theories such as the old friends hypothesis and the microbiome depletion hypothesis (209, 210). From an ecological perspective it seems plausible that the loss of diversity, and in particular the loss of certain linages of microbes, could have negative implications for human health. This notion has the broad support of the World Allergy Organization, who point to the “mounting evidence” suggesting alterations in microbiota correlate with inflammatory disease (211).

It should also be noted that the original hygiene hypothesis was developed when we had a lesser appreciation of the importance of non-communicable diseases in low-income (“less-hygienic”) settings. Diseases such as asthma have likely been overlooked in low-income settings for decades, in part because the burden of asthma has been less than, for example, the burden of acute respiratory infections. Illustrating this point is that asthma was one of the top 10 contributors to death (for both children and all-ages) in Papua New Guinea in both 2007 and 2017 (http://www.healthdata.org/papua-new-guinea). While there was an apparent increase in the burden of asthma between 2007 and 2017, this probably reflects the decrease in burden of diarrhoeal disease during the same period. While not discounting the role of exposure and carriage of either commensal or pathogenic microbes in the risk of non-communicable diseases in the Pacific or elsewhere, further work is required to elucidate the merit and mechanisms of such hypotheses. As raised previously, such work should go beyond investigating the role of bacteria to include other microorganisms as well, a point also raised by World Allergy Organization (211). In brief, there is much to learn from furthering our understanding of interactions between the microbiota of Pacific people and immune diseases. Such studies should derive benefit for Pacific people, for which asthma and other immune diseases are a significant health problem; but may also inform our global understanding of these diseases.

Probiotics have long been recognized as potentially beneficial to human health, with a focus in recent decades on their role as immunomodulatiors (212). This has also lead to a focus on dietary components that support the growth of probiotics and related, perceived beneficial taxa. The role of indigenous fermented foods, generally a rich source of probiotics, varies among the different Pacific cultures; but in summary their traditional role appears less than in Asia and Europe. In lowland and coastal PNG sago starch undergoes traditional fermentation (137), and breadfruit fermentation has been documented in the South Pacific (213); however, other fermentations in the region are seldom documented. It is likely that the traditional diet (section Indigenous Diets in the Pacific and the Transition to a Western diet), which is rich in what are now referred to as prebiotics, may impact on “gut health” and immunomodulation; though no correlative or experimental studies have been conducted specifically relating to Pacific people.

### Gut Microbiome Interaction With Gastrointestinal Pathogens

Much of the global focus of gut microbiome studies has been on their interactions with non-communicable diseases. However, given the ongoing high burden of infectious diseases in low-income settings the interactions between the gut microbiome and pathogens should not be overlooked. Soil-transmitted helminths (STH) infect 2 billion people, and remain endemic in the majority of developing countries (214, 215). The most predominant organisms are the roundworms *Ascaris lumbricoides*, hookworms such as *Necator americanus* and *Ancylostoma duodenale*, and whipworms *Trichuris trichiura*, which heavily impact human health, and children in particular, via impairing nutrition and suppressing host immunity (215–217). Where data are available it appears that the burden of STHs is high in Pacific countries (218–221). It is estimated that 5.5 million people are infected with hookworm and 1.2 million people are infected with *Ascaris* sp. in Oceania, comprising ~1% of the global burdens for both of these infections (222–224).

Gastrointestinal protozoan infections are likely prevalent in the Pacific, although data are scarce. A large-scale surveillance of gastrointestinal protozoa in school children in Fiji sought to detect gastrointestinal protozoa. When detection was by PCR, the prevalence of *Giardia* was 22% and *Entamoeba histolytica* was 2.3% (225). A small study in pregnant women in the highlands of Papua New Guinea revealed high burdens of *E. histolytica* (43%) and *Giardia* (39%) (221). E. histolytica has also been detected in patients with hepatic abscesses in New Caledonia (226). The lack of data may well misrepresent the true burden of protozoan diseases in the Pacific. These parasites are likely to have important direct health impacts on people of the region, and may interact with the gut microbiome and immunoregulation.

Parasites are largely reported for their immunomodulatory properties to evade the host's immune system, which may as a consequence impact on the host's ability to respond to other pathogens (227). Chronic STH infection have been linked to decreased vaccination efficacy and increased susceptibility to co-infection (228, 229), suggesting that helminths—microbiome interactions need to be taken into consideration when envisaging immune-related intervention strategies in endemic areas.

Although the causality between parasitic infection, the microbiome and immune fitness has yet to be fully determined, it may be that such infections have a positive impact on human health. Iatrogenic infection with *N. americanus* was shown to promote species richness and evenness, and increase the production of fecal SCFAs (191, 224, 230). Similarly, it has been hypothesized that gastrointestinal protozoa may have a positive impact on the gut microbiome (231).

A potential role of the gut microbiome in susceptibility and/or resistance to other gastrointestinal pathogens such as bacterial and viral organisms has been raised, but is yet to be fully elucidated. Basic ecological principles suggest that a healthy and well-established gut microbiota will impede colonization by transient gastrointestinal pathogens (232, 233). However, the impacts are not limited to just the microbiota, and the microbiome and associated metabolomics characteristics are likely important factors in the probability of colonization, as is the case with gastrointestinal parasites (above). Bacterial gastrointestinal pathogens appear to be a major contributor to diarrhea in PNG, with shigellosis being an ongoing problem (234–236), and cholera being a sporadic problem (237). The high asymptomatic carriage of bacterial and viral diarrhoeal pathogens (238) in Papua New Guinea may be indicative of low levels of sanitation and hygiene, but nonetheless warrants further investigation in relation to immunity and gut microbiome function.

## Conclusions

The indigenous people of Oceania currently face a variety of health challenges including a high burden of infectious and non-communicable diseases. Both of these are likely to be exacerbated by the ongoing climate change crisis in the region. The Pacific islands are among the most vulnerable to the impacts of climate change and resulting sea-level rise and changing weather patterns (239). Due to the limited health and basic service capacities in many of these countries they will be faced with considerable challenges to rapidly adapt and manage these risks. Alongside social and environmental determinants of health—genetic and microbiotic factors need to be considered and further investigated to ensure improved health outcomes for people in the region. Indeed, the inclusion of indigenous people from that Pacific in studies investigating conditions where indigenous populations may exhibit increased vulnerability (e.g., influenza, obesity, T2DM), or conversely increased resistance (e.g., severe dengue), may be important for global populations.

## Author Contributions

PH, AT, CG, MM, EK, MU, SN, and AG contributed to drafting the manuscript. The final manuscript was reviewed and approved by all authors.

### Conflict of Interest Statement

The authors declare that the research was conducted in the absence of any commercial or financial relationships that could be construed as a potential conflict of interest.
